# The Value of Shunt Surgery or Prophylactic Antiepileptic Therapy or Both in the Development of Dementia at Early Stages in Patients With Ventricular Dilatation

**DOI:** 10.7759/cureus.25423

**Published:** 2022-05-28

**Authors:** Konstantinos Faropoulos, Ourania Fotakopoulou, George Fotakopoulos

**Affiliations:** 1 Neurosurgery, Nicosia General Hospital, Nicosia, CYP; 2 Pediatrics, General Hospital of Zakynthos "Agios Dionysios", Gaitani, GRC; 3 Neurosurgery, General University Hospital of Larissa, Larissa, GRC

**Keywords:** inph, shunt surgery, antiepileptic therapy, ventricular dilatation, dementia

## Abstract

The primary purpose of the current study was to determine the value of the shunt surgery and/or prophylactic antiepileptic therapy, in patients after mild traumatic brain injury (mTBI) with ventricular dilatation (VD) and incipient cognitive impairment, in the prevention of cognitive deterioration and probably in the development of dementia.

Based on the following criteria: a) mTBI b) VD detected in CT scan during admission, and c) the presence of one of the following: i) dizziness, ii) headache, and iii) seizures, admitted to the Emergency Department between January 2010 and January 2020, we enrolled 127 of 947 eligible subjects.

The subjects were divided into five groups: Group A (control group): only VD illustration in CT scan, Group B: incipient dementia, who had a more insidious onset presenting with cognitive dysfunctions at indefinite ages, Group C: shunt system (SH)/antiepileptic drugs (AEDs) presenting with cognitive dysfunction and urinary incontinence or gait disturbances or both, that were treated as idiopathic normal-pressure hydrocephalus (iNPH) with the surgical placement of an SH and AED therapy (standard AED phenytoin (1000 mg loading dose followed by 300 mg) daily), and Group D: AED, presenting with cognitive dysfunctions at indefinite ages and one or two episodes of seizures in the past, treated with AED from the very first moment of initiation with a standard AED phenytoin (1000 mg loading dose followed by 300 mg) daily.

Overall, improvement in daily activities was achieved in 14.1% (18 of 127 patients), recording a significantly higher performance in group D (5.5%) rather than in groups A (1.5%), B (3.1%), and C (3.9%), (p < 0.05). We concluded that changes in VD (ΔVD) were associated with improvement in mRS (ΔmRS ≥ 1) - daily activities and mental status. ΔVD was also independently associated with reduced daily activities during the long-term follow-up. Interestingly, therapeutic shunting and AED in patients with a history of epilepsies may have a positive impact on the development of mental status impairment. This is a novel observation that has to be confirmed by more extensive multicenter studies in the future.

## Introduction

Dementia is a syndrome of behavioral modification and cognitive disturbance caused by diffuse cerebral dysfunction and can be focal as well, such as in primary progressive aphasias [1]. Epidemiological studies demonstrate that 4-12% of the general population over the age of 65 years have some form of dementia [2]. Although there are many types of dementia, each with its own unique characteristics and pathology with distinct behavioral and radiological findings, two forms are most commonly recognized: Alzheimer's type dementia and cerebral arteriosclerosis-related dementia [3]. Clinical recognition of findings in both types of dementia is usually slow, and it is not easy to differentiate between them [4].

The early and exact diagnosis of dementia is valuable since there is the possibility of treating underlying diseases. In the literature, there is evidence that proposes that seizures [5,6] or ventricular dilatation (VD) [7] can accompany dementia in earlier stages more commonly.

In spite of widely varied estimates, seizures are known to occur more frequently in patients with dementia than in the general population [8,9]. They are able to escape detection since they are often non-motor in character, and the history given particularly by aged patients with dementia is frequently either unreliable or unobtainable [10]. Therefore, antiepileptic drugs (AEDs) have been the focus of significant attention as an alternative to existing management. On the other hand, there is a paucity of detailed research assessing the use of AEDs in patients with dementia [2].

Cerebral VD, including the enlargement of ventricular volume and longitudinal ventricle changes, occurs in mild cognitive impairment and is accelerated in dementia [7]. Moreover, the use of cerebral ventricular volume as a measure of the progression of dementia is supported by several studies [11,12]. Ventricular changes such as VD in dementia correlate with extrapyramidal signs [13] but have not been inspected as a precursor of dementia, despite its involvement with axial motor impairment [14] and age [15]. Notably, patients with progressive dementia and bilateral symmetrical VD were considered to suffer from a pathologic condition similar to idiopathic normal-pressure hydrocephalus (iNPH) [16]. Shunt surgery can be performed for these conditions but with very low effectiveness [16] since the outcome of such cases depends on the reversibility of any abnormality of the cerebral parenchyma, such as atrophy [17].

The primary purpose of the current study was to examine the value of shunt surgery and/or prophylactic antiepileptic therapy (AEDs) in the prevention of cognitive deterioration and development of dementia in patients with VD and incipient dementia.

## Materials and methods

This was a retrospective study carried out in Nicosia General Hospital, Department of Neurosurgery, Nicosia, Cyprus on patients presenting in the Emergency Department between January 2010 and January 2020. From 1762 patients with mild traumatic brain injury (mTBI) (Glasgow Coma Scale (GCS) 15-14) based on the classification in Table 1 [18], dizziness, headache, or seizures without any other symptoms, and after CT scan, we found a total of 127 patients with VD. These 127 eligible subjects with VD were divided into four groups: (1) Group A: VD (control), with only VD illustration in CT scan, (2) Group B: Dementia, with incipient dementia, who had a more insidious onset presenting with cognitive dysfunctions at indefinite ages, (3) Group C: shunt system (SH)/AED, presenting with cognitive dysfunction and urinary incontinence or gait disturbances or both, that were treated as iNPH with the surgical placement of an SH and AED therapy (standard AED phenytoin (1000 mg loading dose followed by 300 mg) daily), and (4) Group D: AED, presenting with cognitive dysfunctions at indefinite ages and one or two episodes of seizures in the past, treated with AED from the very first instance of admission with a standard AED phenytoin (1000 mg loading dose followed by 300 mg) daily.

**Table 1:** Classification of TBI severity TBI: traumatic brain injury, PTA: patients with post-traumatic amnesia, GCS: Glasgow Coma Scale, CT: computer tomography, LoC: Loss of consciousness Source: [18] used with permission from Mary Ann Liebert Inc.

Criteria	Mild TBI	Moderate TBI	Severe TBI
GCS score	15-13	12-9	8 - 3
PTA	0-1 days	1-7 days	> 7 days
LoC	< 30 min	30 min-24 hours	> 24 hours
Abbreviated Injury Scale score (Head)	1-2	3	4-6
CT Imaging	Normal	Normal /Abnormal	Normal / Abnormal

Exclusion criteria

Patients younger than 18 and older than 85 years (18 < x < 85 ), with a medical history of alcohol or drug consumption, mental retardation or central nervous system (CNS) pathology, and cognitive disorders before TBI were excluded.

Psychometric assessments

Psychometric assessments were performed both at baseline and at 1-10 years during follow-up for all patients. Although there were several tests carried out on patients with dementia, two of these tests were chosen for this study based on their use in multicenter studies:

(A) The Diagnostic and Statistical Manual of Mental Disorders, Fifth Edition, criteria were used for the diagnosis of dementia [19]: i) memory impairment, ii) at least one of the following: aphasia, apraxia, agnosia, and disturbance in executive functioning, iii) the cognitive deficits cause significant impairment in social or occupational functioning and represent a significant decline from a previous level of functioning, and iv) the cognitive deficits do not occur exclusively during the course of delirium. CNS pathology was determined through medical history, alcohol consumption was assessed using the Alcohol Use Disorders Identification Test-Consumption [20], and drug consumption was assessed via drug abuse testing from a 24-hour timed urine or serum sample [21].

(B) The mini-mental test (mMT) was used for the investigation of cognitive deﬁcits; ﬁrst, as a prescreening test during the admission [22], and during the follow-up. All patients evaluated with electroencephalography, MRI, or CT scan, and with a usual neurological examination for 1-10 years follow-up, including the GCS, were assessed (performed by two physicians other than the treating physicians). In all patients, a measurement of changes in the longitudinal VD (0-3; mean), changes in mMT (ΔmMT), and the modified Rankin scale (mRS) was performed and used to measure performance in activities of daily living.

Outcomes

The primary endpoint was an improvement at ≥ 1 level in the mRS [23] at 1-10 years during follow-up (favorable outcome). The secondary outcome measures included: a) changes in mMT (ΔmMT). Patients were scored both on the day of admission (mMT0) and 1-10 years during follow-up (mMT1). Changes in cognitive function were expressed with the interval between two trial phases (ΔmMT = mMT1-mMT0); b) Changes in VD (ΔVD). After performing CT-scans, the longitudinal ventricular measurement on the day of admission VD0, and in 1-10 years, during follow-up (VD1) was performed. Changes in ventricular measurement were expressed with the interval between the two trial phases [ΔVD = VD1-VD0]. c) Cognitive Deterioration.

Definitions

The mRS measures the performance in daily activities. The ΔmRS was calculated as the difference between baseline (mRS0, day of hospital admission due to TBI) and follow-up (mRS1) (ΔmRS = mRS1-mRS0). We considered ΔmRS as an index of the cognitive impairment due to progressive memory disturbance that may lead to difficulties in learning new things, concentrating, or making decisions in the everyday life [24]. 

To calculate ΔmMT, patients were assessed at admission (mMT0) and follow-up (mMT1), (ΔmMT = mMT1-mMT0). ΔmMT values ≥ 3 were considered an improvement in mental status. ΔVD or Evans' index (EI) > 0.3 was assessed by the longitudinal ventricular on the baseline CT scans (day of admission - VD0) and the follow-up CT (VD1) (ΔVD = VD1-VD0). Signs and symptoms of cognitive disorder were the presenting cognitive dysfunction and urinary incontinence or gait disturbances or both as assessed by mMT, mRS.

Therapy by SH and AED were both retrieved from patients' medical records.

Clinical classifications

Patients were classified into four groups based on clinical symptoms, signs of cognitive disorder, and management (shunt surgery or/and AED). Hence they were classified into Group A: patients without symptoms/signs of cognitive disorder or therapy; Group B: patients with symptoms/signs of cognitive disorder; Group C: patients with symptoms/signs of cognitive disorder, at least one documented seizure episode of epilepsy and AED; Group D: patients with symptoms/signs of cognitive disorder, at least one documented episode of epilepsy and AED and SH treatment; Group E: patients with symptoms/signs of cognitive disorder, at least one documented episode of epilepsy and SH but without AED.

Statistical analysis

Continuous data are expressed as mean (±SD). Data were assessed for normality using the Shapiro-Wilkes test. Nominally distributed data were analyzed using Fisher’s exact test. Continuous data were analyzed using Student’s t-test or Mann-Whitney U-test as appropriate. Variables significantly associated during univariate analysis were then entered into a multivariable analysis model. Values of p < 0.05 were considered statistically significant. Statistical analyses were performed with SPSS for Windows, Version 15.0 (Released 2007; SPSS Inc., Chicago, United States).

## Results

A total of 947 patients were screened and 127 patients were included in this study. The baseline characteristics of the study participants are shown in Table 2. Statistically significant differences between the groups were found according to the cause of admission (p < 0.05), urinary incontinence (p < 0.05), and gait disturbance (p < 0.05).

**Table 2:** Baseline characteristics of patients Data are presented as mean ±SD, otherwise is indicated; P-value for the difference between groups was assessed for nominal data using the Fisher’s exact test and for continuous data with the Mann-Whitney U test as appropriate. mTBI: mild Traumatic Brain Injury

Parameters	Group A: n=40 (31.4%)	Group B: n=60(47.4%)	Group C: n=13(9.8%)	Group D: n=14(11.5%)	P-value
Age, years	68.8±7	69.2±6	66.1±7	72.0±5	0.232
Sex (Male), n(%)	20(15.7)	30(23.6)	7(5.5)	7(5.5)	0.995
Cause of admission					
-mTBI or Seizures,n(%)	27(21.2)	32(25.1)	13(10.2)	14(11.0)	0.000
-others, n(%)	13(10.2)	28(22.0)	0(0)	0(0)	
Hypertension, n(%)	16(12.5)	25(19.6)	4(3.1)	6(4.7)	0.902
Coronary heart disease, n(%)	16(12.5)	26(18.8)	5(3.9)	8(6.2)	0.707
Chronic smokers, n(%)	13(10.2)	20(15.7)	4(3.1)	7(5.5)	0.643
Diabetes, n(%)	14(11.0)	19(14.9)	3(2.3)	7(5.5)	0.483
Urinary incontinence, n(%)	3(2.3)	8(6.2)	0(0)	9(7.0)	0.000
Gait Disturbance, n(%)	3(2.3)	4(3.1)	4(3.1)	10(7.8)	0.000

Outcomes

Clinical outcomes across groups are shown in Table 3.

**Table 3:** Patients’ outcomes Data are presented as mean ±SD, otherwise is indicated: P-value for the difference between groups was assessed for nominal data using the Fisher’s exact test and for continuous data with the Mann-Whitney U test as appropriate mRS: modified Rankin scale; VD; ventricular dilatation; mMT: mini-mental test; ΔmRS interval between two trial phases, on the day of admission (mRS0) and 1–10 years during follow-up (mRS1); ΔVD: interval between two trial phases of ventricular dilatation measurement (VD1-VD0); VD0: measurement on the day of admission (baseline);  VD1: testing 1-10 years during follow-up;  ΔmMT: interval between two trial phases of mini-mental test measurement (mMT1-mMT0); mMT0: testing took place on day of admission (baseline); mMT1: testing took place 1-10 years during follow-up

Parameters	Group A: n=40 (31.4%)	Group B: n=60 (47.4%)	Group C: n=13 (9.8%)	Group D: n=14 (11.5%)	P
ΔmRS, mean±SD	0.05±0.2	0.07±0.2	0.38±0.5	0.50±0.5	0.000
ΔVD, mean±SD	0.97±0.1	0.98±0.1	0.08±0.2	-1.1±0.5	0.000
ΔmMT, mean±SD	-2.53±1.5	-2.4±2.1	3.6±2.4	2.8±2.4	0.000

ΔmRS ≥ 1 was achieved in 14.1% (18 of 127 patients) recording a significantly higher performance in group D (5.5%) compared to group A (1.5%), B (3.1%), or C (3.9%) (all p < 0.05). ΔmRS was significantly associated with ΔVD for subjects of group D (p < 0.05) (Table 4).

**Table 4:** Patients' mRS score P-value for the difference between groups was assessed for nominal data using the Fisher’s exact test and for continuous data with the Mann-Whitney U test as appropriate mRS: modified Rankin scale; ΔmRS: interval between two trial phases, on the day of admission (mRS0) and 1–10 years during follow-up (mRS1)

Parameters	Group A: n=40 (31.4%)	Group B: n=60 (47.4%)	Group C: n=13 (9.8%)	Group D: n=14 (11.5%)	P
Primary					
ΔmRS, n (%)					
≥1	2(1.5)	4(3.1)	5(3.9)	7(5.5)	0.000
<1	6(4.7)	23(18.1)	8(6.2)	7(5.5)	0.040

Group, gait disturbance, and ΔVD were associated with improvement in mRS (ΔmRS ≥ 1) (p < 0.05). Multivariate analysis revealed that ΔVD (p < 0.05) was the only independent risk factor for the improvement (ΔmRS ≥ 1) (Table 5).

**Table 5:** Independent risk factors of improvement of patients after multivariable analysis P-value for the difference between parameters was assessed for nominal data using the Fisher’s exact test and for continuous data with the Mann-Whitney U test as appropriate. mRS: modified Rankin scale; mMT: mini-mental test; ΔVD: interval between two trial phases of ventricular dilatation measurement (VD1-VD0); VD0: measurement took place on day of admission (baseline); VD1: testing took place 1-10 years during follow-up; CI: conﬁdence interval; OR: odd ratio

Name	OR		95%CI (lower-upper)		P
According to mRS					
Groups	0.026	-0.049- 0.100		0.497	
Gait Disturbance	-0.078	-0.252-0.096		0.377	
ΔVD	-0.254	-0.361-(-0.146)		0.000	
According to mMT					
Groups	0.181	0.118-0.243		0.000	
mTBI or Seizures	0.023	-0.076-0.121		0.651	
Gait Disturbance	-0.002	-0.148-0.143		0.978	
ΔVD	-0.124	-0.216-(-0.033)		0.008	

**Figure 1:**
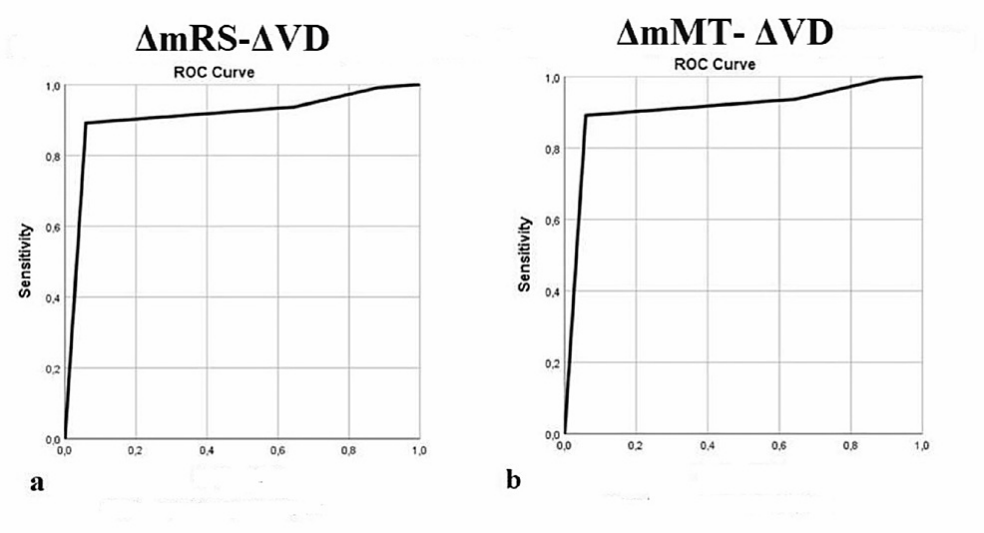
Scatter plots (a) A scatter plot of the association between changes in ventricular dilatation (ΔVD) and changes in score on the modified Rankin scale (ΔmRS) for daily activities. A decline in the mRS < 1 score suggests a decline in daily activities. (b) A scatter plot of the association between changes in ventricular dilatation (ΔVD) and changes in score on the mini-mental exam (ΔmMT) for mental status. A decline in mMT < 3 score suggests a decline in mental status.

Receiver operating characteristic (ROC) analysis showed that ΔVD was the most accurate measure for identifying improvement in daily activities according to mRS (ΔmRS ≥ 1) with the area under curve standard error (AUC(SE)) of 0.830 (0.060), p < 0.05 (Table 6), whereas a ΔVD value of 0.5 presented with 94.5% sensitivity and 61.1% specificity for improvement in daily activities (Figure 1).

**Table 6:** Statistical ﬁndings for ROC P-value for the difference between parameters was assessed for nominal data using the Fisher’s exact test and for continuous data with the Mann-Whitney U test as appropriate ROC: receiver operating characteristic; CI: conﬁdence interval; mRS: modified Rankin scale; mMT: mini-mental test; ΔmRS: interval between two trial phases, on the day of admission (mRS0) and 1–10 years during follow-up (mRS1); ΔmMT: interval between two trial phases of mini-mental test measurement (mMT1-mMT0); mMT0: testing took place on day of admission (baseline); ΔVD: interval between two trial phases of ventricular dilatation measurement (VD1-VD0); VD0: measurement took place on day of admission (baseline); VD1: testing took place 1-10 years during follow-up; Std Error=standard error

Parameters	Area	Std Error	CI(95%) lower-upper	P
ΔmRS –ΔVD	0.830	0.060	0.711-0.948	0.000
ΔmMT –ΔVD	0.907	0.038	0.833-0.982	0.000

mMT

The ΔmMT across the groups is shown in Table 3. Group D presented greater cognitive decline according to the ΔmMT (p < 0.05) when compared with the other groups (Table 3). Univariate analysis revealed that group (p < 0.05), cause of admission (mTBI or Seizures) (p = 0.012), gait disturbance (p < 0.05) and ΔVD (p < 0.05) were significantly associated with improvement in mental status (ΔmMT values ≥ 3) (Table 7).

**Table 7:** Univariate analysis for ΔmRS and ΔmMT Data are presented as mean ±SD, otherwise is indicated; P-value for the difference between groups was assessed for Nominal data using the Fisher’s exact test and for Continuous data with the Mann-Whitney U test as appropriate mRS: modified Rankin scale; VD: ventricular dilatation; mMT: mini-mental test; ΔVD: interval between two trial phases of ventricular dilatation measurement (VD1-VD0); VD0: measurement took place on day of admission (baseline); VD1: testing took place 1-10 years during follow-up

Parameters	ΔmRS			ΔmMT		
	≥ 1 n=18 (14.1%)	< 1 n=109(85.9%)	P	≥ 3 n=17(13.3)	< 3 n=110 (86.7)	P
Groups						
-Group A: n (%)	2(1.5)	38(29.9)	0.000	0(0)	40(31.4)	0.000
-Group B: n(%)	4(3.1)	56(44.0)		1(0.7)	59(46.4)	
-Group C: n(%)	5(3.9)	8(6.2)		6(4.7)	8(6.2)	
-Group D: n(%)	7(5.5)	7(5.5)		10(7.8)	3(2.3)	
Age, years	69.39±7.5	69.07±6.4	0.755	69.82±7	69.01±6	0.351
Sex (Male), n(%)	9(7.0)	55(43.3)	0.971	9(7.0)	55(43.3)	0.821
Cause of admission						
-mTBI or Seizures,n(%)	15(11.8)	71(55.9)	0.126	16(12.5)	70(55.1)	0.012
-others, n(%)	3(2.3)	38(29.9)		1(0.7)	40(31.4)	
Hypertension, n(%)	6(4.7)	45(35.4)	0.524	5(3.9)	46(36.2)	0.331
Coronary heart disease, n(%)	7(5.5)	48(37.7)	0.683	5(3.9)	46(36.2)	0.331
Chronic smokers, n(%)	6(4.7)	38(29.9)	0.899	7(5.5)	37(29.1)	0.543
Diabetes, n(%)	5(3.9)	38(29.9)	0.556	6(4.7)	37(29.1)	0.893
Urinary incontinence, n(%)	5(3.9)	15(11.8)	0.130	4(3.1)	16(12.5)	0.334
Gait Disturbance, n(%)	7(5.5)	16(12.5)	0.006	8(6.2)	13(10.2)	0.000
ΔVD, mean±SD	-0.28±0.9	0.81±0.5	0.000	-0.41±0.7	0.82±0.5	0.000

Multivariate analysis revealed that Group (p < 0.05) and ΔVD (p = 0.008) were independent risk factors for the improvement in mental status (ΔmMT ≥ 3) (Table 5). ΔVD was the most accurate measure for identifying improvement in mental status with AUC(SE) of 0.907 (0.038), p<0.05 (Table 6), whereas a ΔVD value of 0.5 presented with 88.9% sensitivity and 41% specificity for improvement in mental status (Figure 1).

## Discussion

The main findings of this study that included patients with a mild head injury and VD were: AED administration and/or SH were effective for the treatment of VD and dementia at the early stages; patients with cognitive dysfunctions and one or two episodes of seizures in the past treated with AED (group D), and patients with cognitive dysfunctions at indefinite ages, urinary incontinence, and gait disturbances that were treated as iNPH with the surgical placement of SH combined with AED therapy (group C) have a better outcome than in those of group B; cognitive dysfunction was an independent risk factor for dementia in patients with VD; the ΔmRs and ΔmMT were significantly associated with ΔVD for subjects of group C or D.

Several studies have reported that the relative risk of seizures in Alzheimer’s disease was 6.6 times higher than in patients without dementia, and the epileptic activity was relative to the early onset of cognitive decline [5,25].

In these patients, cognitive dysfunction seems to be recovered after AED treatment [26]. However, conflicting reports mentioned that AEDs were not helpful for such patients and were associated with a high risk of adverse effects, particularly visual disturbance, mental slowing, confusion, and ataxia [27]. If this cognitive dysfunction was associated with epilepsy, it should be considered at the time of dementia diagnosis. Poorly controlled epilepsy can cause cognitive dysfunction by virtue of abnormal brain activity. Cognitive dysfunction does not equate to dementia. In our study, cognitive dysfunction was an independent risk factor for dementia in patients with VD. Patients that suffered from VD and cognitive dysfunction, without episodes of seizure in the past nor gait disturbance/urinary incontinence and were not treated with AEDs plus SH seemed to have a poorer outcome, as they showed a progressive deterioration.

A study assessed the ventricular changes in subjects with dementia and found that regional ventricular enlargement was associated with disease progression [28]. Furthermore, MRI studies in dementia have measured, among others, the volumes of brain structures, concluding that they might be potential biomarkers of the progression of Alzheimer’s disease, helping to identify factors that either speed up or slow down brain degeneration, either in clinical trials or genetic studies on dementia [28]. A larger ventricular size represents a susceptibility factor for dementia or a marker of dementia-related pathology in subjects with rapidly degenerating brains [28]. In our study on patients with VD who presented with cognitive dysfunction and one or two episodes of seizure in the past with or without urinary incontinence/gait disturbances and treated with AEDs or SH or both, the cognitive improvement correlated with the proportionate ventricular volume reduction.

In the literature, ventricular expansion has been implicated in the development of Alzheimer’s disease, Parkinson’s disease, and INPH [16].

VD and the rate of change of its volume have been associated with future cognitive decline and dementia [29]. Furthermore, a higher rate of VD reflected both white and gray matter loss in patients experiencing incident dementia or significant cognitive decline [30]. Our study demonstrated that ventricular changes (ΔVD) were associated with cognitive modifications (ΔmMT) and the improvement in daily activities according to mRS (ΔmRS ≥ 1) in patients with one or two episodes of seizure in the past with or without urinary incontinence/gait disturbances that were previously treated as iNPH with the surgical placement of SH or AED administration or both.

One limitation of our study was its small sample size, which restricted our power to achieve statistical significance in several of the analyses. Moreover, the mRS was one of the most accurate tests for dementia’s development/progression especially at the early stages. However, larger studies presently underway will provide the necessary statistical power to detect or refute potential between group differences. The probability from the ex vacuo dilatation of the ventricles with the normal aging and education variance observed between the groups was another relative limitation.

## Conclusions

We concluded that AED or SH administration or both were effective for the treatment of VD accompanied by dementia during the early stages. The risk of seizures in this population was high; therefore, physicians may begin treatment with AEDs after a single seizure if there is focal neurological involvement or if there is any concern that seizures may recur. Also, in those subjects, SH therapy may be considered, especially if the cause of the urinary incontinence/gait disturbances that the patient presented with was not identified after a thorough clinical history was taken and a detailed neurological examination was performed. Given our small sample size and heterogeneous cognitive outcomes, this requires further validation.
